# Psychosis seminars: an open forum for service users, carers and professionals

**DOI:** 10.1192/bjpo.bp.116.003269

**Published:** 2016-10-28

**Authors:** Justina Kaselionyte, Aysegul Dirik, Simon Tulloch, Stefan Priebe, Domenico Giacco

**Affiliations:** **Justina Kaselionyte**, MSc, Unit for Social and Community Psychiatry (WHO Collaborating Centre for Mental Health Service Development), Newham Centre for Mental Health, Queen Mary University of London, London, UK; **Aysegul Dirik**, MSc, Unit for Social and Community Psychiatry (WHO Collaborating Centre for Mental Health Service Development), Newham Centre for Mental Health, Queen Mary University of London, London, UK; **Simon Tulloch**, MSc, Quality Outcomes and Experience, East London NHS Foundation Trust, London, UK; **Stefan Priebe**, FRCP, Unit for Social and Community Psychiatry (WHO Collaborating Centre for Mental Health Service Development), Newham Centre for Mental Health, Queen Mary University of London, Newham Centre for Mental Health, London, UK; **Domenico Giacco**, PhD, Unit for Social and Community Psychiatry (WHO Collaborating Centre for Mental Health Service Development), Newham Centre for Mental Health, Queen Mary University of London, London, UK

## Abstract

**Background:**

Psychosis seminars enable service users, their carers and mental health professionals to meet outside of a formal care setting, increase understanding of mental illness and help establish a dialogue.

**Aims:**

To explore feasibility of psychosis seminars in the UK and the experiences of participants.

**Method:**

Seven meetings attended by 25 people were held over a 3-month period. An open-ended questionnaire was returned by ten participants. Responses were subjected to content analysis.

**Results:**

Benefits experienced were having an open forum for talking freely about mental health issues in a neutral space, learning from others about psychosis and hearing different views. Suggested adjustments were clarifying expectations of participants at the beginning, strengthening facilitation and increasing attendance.

**Conclusions:**

Psychosis seminars may help to establish a dialogue among users, carers and professionals and seem feasible in the UK, although adjustment to delivery can help their implementation.

**Declaration of interest:**

None.

**Copyright and usage:**

© The Royal College of Psychiatrists 2016. This is an open access article distributed under the terms of the Creative Commons Attribution (CC BY) license.

In recent years, user and carer participation in the development of mental healthcare and expertise has come into sharper focus.^1^^–^^3^ This has contributed to improving mental health services in terms of their accessibility and diversity^4^ and increased the quality of staff training and service evaluation endeavours.^5^^,^^6^ Service users and carers seem to benefit from a more active role too by learning new skills, increasing their confidence, sense of empowerment, social inclusion and developing better relationships with mental health professionals.^5^^,^^7^^–^^9^ However, the efforts to establish a constructive dialogue and form trusting relationships between service users, carers and professionals are often hindered by negative attitudes towards each other and power issues.^8^^,^^10^^,^^11^ Different perspectives, interests and terminologies may make communication difficult and prevent individuals from exchanging their experience and knowledge of mental health.^11^^,^^12^

Psychosis seminars offer an innovative form of communication and interaction among service users, carers and professionals.^12^ The aim of these seminars is to discuss the experiences of mental health difficulties and the ways to dealing with them.^13^ According to Bock & Priebe,^12^ psychosis seminars do not provide a form of medical treatment, but a ‘mutually respectful dialogue’ and the opportunity for the participants to meet ‘with equal entitlements and without any formal responsibilities’ (p. 1441). Participation in these seminars may help in modifying the attitudes of professionals, carers and service users towards each other and stimulate a constructive dialogue. This may, in turn, facilitate communication and collaboration between the three groups in service settings.^14^

According to the psychosis seminars model, meetings are held regularly after traditional office hours in a neutral environment, which does not include a therapeutic or familial context, and can be attended by all interested service users, carers and mental health professionals. Each meeting focuses on a particular topic, which is selected by the participants.^12^

Psychosis seminars originated from the collaboration between a psychiatric survivor of the German Nazi regime Dorothea Buck, psychologist Thomas Bock and journalist Ingeborg Esterer.^14^ The first psychosis seminar was organised in 1990, in Hamburg, Germany.^12^ Their successful example was soon followed by Austria and Switzerland establishing similar ‘trialogical’ meetings.^15^ Since then, the model has been increasingly adopted in other countries such as Poland, Turkey, Lichtenstein and Ireland^15^^,^^16^ and has been described as an example of successful trialogue between service users, carers and mental health professionals.^2^^,^^14^ In the UK, the psychosis seminar model has not yet been implemented.

In other countries in which psychosis seminars were organised, for example, Ireland and Germany, provision of care is funded through personal health insurance, whereas in the UK the healthcare is funded by government based on taxpayer money. Given the difference in funding and organisation of care, in the UK, there may be different types of challenges for organising independent out-of-hours initiatives outside of regular care provision.

The first attempt to establish psychosis seminars in the UK was made by East London NHS Foundation Trust (ELFT). The aim of this study is to examine the participants’ experiences of psychosis seminars in order to explore whether this model is suitable in the UK or requires adaptation for implementation in this country.

## Method

### Procedure

The project was undertaken as the initiative of a provider of mental health services (ELFT) as a way to increase levels of carer and service user involvement.

Service users, carers and mental health professionals in contact with or working for mental health services across the boroughs of City and Hackney, Newham and Tower Hamlets were invited to participate in the seminars. These boroughs are exclusively inner-city areas with high prevalence rates of severe mental health problems and high levels of deprivation.^17^ A member of the project team (S.T.) contacted all in-patient wards, the day hospital and community mental health teams in ELFT. He attended various meetings, including community meetings on wards and at the day hospital, and presented the project at a range of service user and carer events. Information leaflets and posters were produced and psychosis seminars were advertised on the intranet and via the ELFT newsletter and magazine.

The seminars were held in line with the formal requirements, that is, outside office hours and at a non-hospital venue (town hall), which was close to public transport. Topics to be discussed in the seminars were jointly agreed by the participants. There was a rotation between service users, carers and professionals in chairing the meetings.

At the end of the project, all participants were asked to complete a brief evaluation form containing the following open-ended questions: ‘What attracted you to attend the psychosis seminars?’ ‘If you did not attend meetings, what prevented you from attending?’ ‘If you did attend meetings, please list three things that you found helpful and three things that you did not like?’ ‘Please list three things that you think would improve the seminars, and how these could be achieved’.

### Analysis

Participant responses were subjected to content analysis. This allowed the identification of trends and patterns in the data by distilling words into categories that share the same meaning.^18^^–^^20^ As the participants’ experience of psychosis seminars has not been extensively studied before, an inductive approach was utilised to provide new insights and richer understanding to be developed directly from the data without using preconceived categories.^18^^,^^19^

The steps for inductive content analysis outlined by Elo & Kyngäs^21^ were followed in this study. After familiarisation, authors J.K., A.D. and D.G. independently analysed the data. Open coding was conducted, making notes and headings in the text in order to describe the content. The process of grouping similar codes under themes followed. The identified themes and subthemes were then checked and refined by J.K., A.D. and D.G. This aided conformability, to represent the participants’ responses as accurately as possible and reflect their voice rather than the researchers’.^22^

## Results

Twenty-five people attended the seven seminars held over a 3-month period. This included 15 female and 10 male participants with authors S.T. and S.P. among them. Although the number and the compositions of participants from the three groups varied at each seminar, a ‘core group’ of 10 participants attended most seminars. The cost of the project, including venue hire and refreshments, came to less than £1500.

Qualitative data about the experiences with attending the seminars were returned by 10 (40%) participants, that is, those who regularly attended the seminars. Four of them were mental health professionals, two carers, three service users and one service user who was also a mental health professional. Respondents did not include authors S.T. and S.P.

**Fig. 1:**
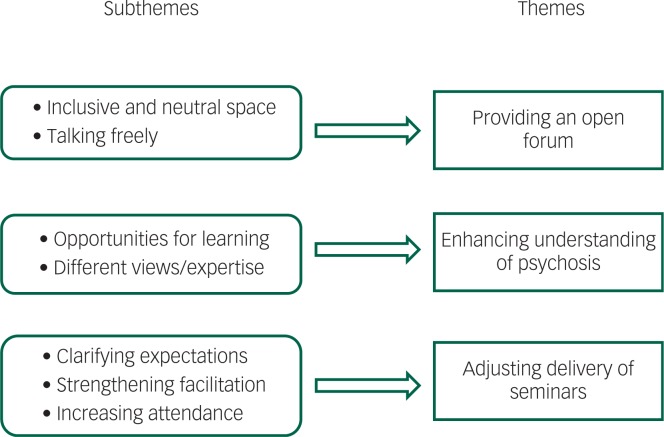
Themes and subthemes.

Three themes were identified in the qualitative data: providing an open forum, enhancing understanding of psychosis and adjusting delivery of seminars. The themes and their subthemes have been illustrated in Fig. 1 and described in detail below. Quotes from the participants’ responses have been used to illustrate the findings and increase the trustworthiness of the results.

### Providing an open forum

This theme emerged from the participant’s feedback about why they attended psychosis seminars and what it was they liked about them. Psychosis seminars as an open forum theme encompassed two elements: the provision of inclusive and neutral space and the opportunity for the participants to talk freely.

#### Inclusive and neutral space

The inclusivity of psychosis seminars, the fact that they were not held in a treatment facility and the opportunity to meet people from different stakeholder groups was valued by participants:

I enjoyed and found it very interesting to meet people from the three groups in a non-hospital environment. (carer 2)

#### Talking freely

Talking freely was an important aspect of the open forum provided by psychosis seminars. For example, one professional stated that they attended the seminars because they wished to have an opportunity for open and free discussion focused on service user and carer concerns. Carers and service users appreciated that psychosis seminars enabled them to share their experiences freely and ask questions to professionals:

[I had the] chance to say my piece. (service user 3).

It was very good that anyone could ask anybody anything and get a reply. (carer 2).

Feeling comfortable to freely share one’s experiences with other people was facilitated by the lack of hierarchy in the groups. It was felt that the open forum was indeed shared by service users, carers and professionals.

One professional mentioned among the aspects that attracted him/her to attend was the

democratic and equal status approach. (professional 8)

### Enhancing understanding of psychosis

#### Opportunities for learning

People were enticed to attend psychosis seminars because they saw them as an opportunity for learning and broadening their horizons:

I want to learn and understand as much as possible about psychosis and available treatment. (carer 2)

Thought that I could learn something about the condition that I could use in future work with myself and others in mind. (service user/professional 6)

It seems a good way to find out what people want in terms of care, what people feel needs to be considered, as well as how psychosis affects people from each of these groups. (professional 10)

Participants reported that they found meetings informative and felt that psychosis seminars helped them to learn more about psychosis and mental health services in general. It seems that participants were indeed keen on learning opportunities as some of them suggested having guest speakers with expertise or experience in mental health at the beginning of each seminar.

#### Different views and expertise

From the outset, participants were interested in learning about others’ experiences at psychosis seminars:

I wanted to get a better understanding of what psychosis is. What it means for those experiencing it, professionals and carers. To help make sense of my experiences (…). (carer 7)

Hearing a variety of perspectives and experiences at the seminars significantly contributed to enhancing participants’ understanding of psychosis. This was commonly noted as one of the most helpful aspects of psychosis seminars:

To hear different experiences, understanding and opinions regarding mental health. (carer 7)

… what I did find really useful was the variety of perspectives. (professional 10)

There was also an acknowledgement of and respect for each other’s expertise. Service users and carers appreciated that professionals were involved in the seminars and professionals benefited from listening to carers and users sharing their experiences:

I thought to have someone professional there was good. (service user/professional 6)

I particularly appreciated the open and clear responses of the professionals. I was interested in the service users’ questions, their knowledge of psychosis and treatment. (carer 2)

Getting sense of service user and carer priorities in a general sense. (professional 8)

### Adjusting the delivery of seminars

The third theme focused on the issues related to delivery of psychosis seminars such as participants’ expectations of the seminars, group facilitation and attendance.

#### Clarifying expectations

Participants reported that they did not know what to expect of psychosis seminars in terms of their format. Although some participants liked the open agenda, others had mixed feelings about it or wished to have some form of structure in place:

The free ranging nature of the meetings did allow interesting things to emerge and too much structure would be a bad thing. (carer 2)

On the one hand I like the spontaneity this arrangement allows. On the other hand, it would be nice to know what to expect when, as some topics may be of more interest than others. (professional 10)

I think for me, if the sessions had been more structured people could benefit from [the] agenda. (service user/professional 6)

Furthermore, it was suggested that ‘shared group expectations’ could be developed as the ground rules were unclear to some of the participants:

Could be clearer on expectations of group members (…) – are we allowed to challenge people, or is the expectation more of a didactic model of presentation and question? (professional 8)

#### Strengthening facilitation

Another subtheme that emerged from the participant’s responses about what could be improved in the delivery of psychosis seminars was the need to strengthen facilitation of groups. Participants felt that there was unequal contribution from people at the seminars with some individuals being more vocal than others. Some people also thought that topics could have been explored in more depth:

Some people talking a lot; some people not getting their fair share of time to say their piece. (service user 4)

Sometimes topics were not discussed fully and I felt there was much more that could have been discussed. (…). Sometimes only a few of the group contributed to discussions and others did not say anything. (carer 7)

Carers dominated the seminar. (professional 9)

Some participants suggested that psychosis seminars could be chaired professionally, which they thought could give more people a chance to share their experiences.

#### Increasing attendance

Finally, participants reported that the number of people attending psychosis seminars was low and suggested that attendance could be increased with better representation from all three groups:

More people from all three groups but particularly service users. (carer 2)

For the next round would it be possible to encourage more even participation of service users, carers and mental health professionals that would involve a senior trainee…and more junior trainees. (professional 1)

The frequency of psychosis seminars being weekly and the requirement to commit to all scheduled meetings seemed to have affected group attendance:

Maybe weekly is too frequent, not sure. (carer 2)

Several of my colleagues had been tempted to join but were put off by the idea of having to commit. (professional 10)

I started and then had to commit to something else and finally, when I did get some time, felt that I might have missed too many [seminars] to continue. (service user/professional 6)

One participant suggested having an open attendance structure to attract more people with a core group who would be recommended to commit to all seminars:

I also think that having an open attendance structure would attract more people. (…) I do, however, also see the value of having a core group which remains constant as that fosters confidence and trust among members. (professional 10)

It was also felt that group schedule and topics could be publicised in advance. Furthermore, one participant thought that the recruitment materials for psychosis seminars could be improved:

The flyer I saw for the seminars was quite busy and wordy. I think this is brilliant for people who want more information, but can be off-putting for those who don’t know anything about it yet. Once I was shown this flyer I realised I had seen it in the reception area of my service – and did not read it for precisely that reason. (professional 10)

## Discussion

### Main findings

In this study, we explored feasibility and participants’ experiences of psychosis seminars, which were held in the UK for the first time. The themes that have been identified in the open-ended questionnaire data captured three aspects of psychosis seminars: the acceptability of the model which provides an open forum for interaction and discussion between service users, carers and professionals, the benefits of attending seminars in terms of learning and understanding more about psychosis and the issues related to adjusting the delivery of the seminars to improve implementation.

Generally, the psychosis seminars model seemed to be well accepted by the participants who reported that they indeed benefited from attending the seminars. This suggests that the seminars may be a promising option to improve communication between users, carers and professionals.

However, there were issues related to the delivery of seminars, including unclear expectations about the format and rules, facilitation issues and low attendance. The original psychosis seminars model has an open agenda that is jointly agreed by the participants in the meeting.^12^ However, in this study, some participants felt that it would have been beneficial to have more structured agenda in advance. Results also suggest that for the future psychosis seminars, participants’ expectations should be clarified. There should be clear explanations on what seminars will entail and how they are going to be run, including some ground rules (e.g. emphasising anonymity, respect and listening to others and priority to subjective perspectives). It would also be possibly helpful to hold an initial meeting where priority topics could be selected.

Furthermore, although psychosis seminars are based on a democratic and equal approach, participants reported unequal contribution of people in the discussions, with some groups dominating the seminar and others not having a chance to speak. This raises questions about how facilitation could be strengthened to ensure that everybody can contribute without undermining the core principles of the equality of psychosis seminars. A suggestion was to have a professional facilitator rather than having a rotating chair from within a group. However, having an external person to run discussions may imply an increase in costs and make the organisation of seminars more complex. Moreover, it is difficult to estimate its effect on the discussion between service users, carers and professionals.

Finally, the number of participants of psychosis seminars was relatively low (25 people) and the participants unanimously reported that more people from each of the three groups would have been beneficial. The attendance seemed to have been affected by the requirement for participants to commit to all weekly seminars in the cycle. This needs to be considered when implementing psychosis seminars in the future. One of the suggestions made by participants was having a core group of people who would attend all seminars and then opening attendance to others on a drop-in basis. A less intensive schedule (e.g. fortnightly meetings) could also be considered.

### Comparison with the literature

The findings of this study are in line with some aspects of the intergroup contact theory, which postulates that equal status, the opportunity to learn about others and the formation of an inclusive group may lead to improved intergroup relations.^23^ Furthermore, the inclusivity and equal status approach in psychosis seminars also fits with the democratisation approach in service user involvement, which encompasses partnership and empowerment of users.^24^

Literature or evaluative studies of psychosis seminars are lacking. Amering *et al*^25^ provided three personal accounts by a service user, a carer and a professional. Their report offers some insights about the opportunities and difficulties that can arise in psychosis seminars such as the chance to freely share opinions and ask questions in a neutral setting, benefits from learning from each other, but also dealing with criticisms from others or difficulties in listening to others’ distressing experiences. Another study collected data using a postal survey of participants of 58 psychosis seminars in Germany and found that all three parties (service users, carers and professionals) benefited from listening to each other without any pressure or responsibility,^12^ which is in accordance with the findings of the present study.

The evaluation of psychosis seminars in Ireland including more than 300 participants (42 of them interviewed) and six sites yielded results consistent with our findings: participants reported that they have learned from each other’s different perspectives on mental health and felt comfortable to speak in an egalitarian meeting, where service users, carers and mental health professionals could listen to each other in a neutral setting.^16^ This confirms the potential of psychosis seminars to facilitate a different form of communication to the one in clinical settings, where different roles and expectations as well as power imbalances and pressure can be found.^14^

Issues related to facilitation of meetings were also reported by Mac Gabhann and colleagues,^16^ with some participants expressing their doubts about their ability to facilitate discussions because of the lack of confidence or skills. Therefore, they decided to have two moderators and developed a set of ground rules and tips on facilitating meetings for participants, which may be a useful resource for adapting delivery of psychosis seminars in the contexts in which issues relating to facilitation arise.

Interestingly, the issues related to the format of seminars such as the lack of a structured agenda reported by the participants in this study were not mentioned in other literature. This raises the question whether in our case this was because of the lack of information to the participants prior to seminars (people not knowing what to expect) or because of something specific to the context, that is, people may have been more used to structured meetings.

### Strengths and limitations

To our knowledge, this is the first study in the UK to explore feasibility and personal experiences of participants in psychosis seminars. Qualitative survey data were collected from all three groups of people who attended the meetings. Content analysis provided a useful framework for analysis in terms of identifying patterns and themes in the data, which helped maximise the information obtained from this exploratory study in an area where systematic research is lacking. Conformability of the analysis was assured by the three authors independently coding the data and refining the themes together. Quotes from the participants’ responses were provided to illustrate the themes and to increase trustworthiness of the results.

The quality of the data collected from the open-ended survey (short or missing responses for some questions) and the small sample (only 25 participants and only 10 of them returned the questionnaire) could be considered as the main limitations of the study. It could be argued that future studies could obtain richer data by the use of in-depth interviews or focus groups with the participants.

Another limitation of our study is that the initiative was professionally led and service users and carers where not involved in the design, delivery or evaluation of the project, which should be considered when future studies are being developed.

It is also important to note that findings could not be generalised to the rest of the UK as psychosis seminars took place only in three boroughs of East London.

### Implications and future research

Psychosis seminars appear to be feasible in the UK and their organisation is not particularly expensive, only requiring room bookings. They may not be an option for all service users, carers or professionals as participation is voluntary and outside working hours.

Yet, they were experienced by the participants positively and could potentially lead to improved communication among service users, carers and professionals during mental healthcare delivery, which is advocated by the government policies but may be hindered by power issues. Our findings also suggest that the way seminars are organised and delivered should be considered carefully and agreed by the group in advance.

Psychosis seminars could be implemented more widely in the UK, following the example and success of the Mental Health Trialogue Network in Ireland as well as well-established psychosis seminars in other countries.

The unconventional nature and philosophy of psychosis seminars may be incompatible with systematic evaluation or experimental trials.^12^ In particular, they are based on voluntary participation and are not strictly speaking part of treatment provision. The effects of participating may be better captured through case studies or mixed-methods observational studies and by assessing changes in attitudes and relationships between service users, carers and professionals.
